# Inefficiency of Thymol as an Antiseptic

**Published:** 1882-05

**Authors:** Edmund Andrews

**Affiliations:** Prof. Clin. Surg. Chi. Med. College; No. 6 Sixteenth St., Chicago


### ARTICLE III.

INEFFICIENCY OF THYMOL AS AN ANTISEPTIC. By Edmund Andrews, A.M., M.D., Prof. Clin. Surg. Chi. Med. College.

Thymol has been very falsely lauded as an antiseptic. Scientific men have ascribed efficiency to it in solutions as weak as one part in five thousand, while others, more careful, said one in two thousand. The test of the surgical power of an antiseptic is its capacity to prevent putrefaction in animal substances. I therefore make the following experiment, to determine approximately what strength of the solution of thymol is required for practical work. The result very much lowered my estimate of this overpraised article, whose only advantage seems to be its agreeable odor.

Taking ten vials, I placed in each two grains of beef muscle, with thirty-two cubic centimeters of fluid of the kinds shown in this table. As pure water will not dissolve the quantity of thymol represented in the stronger solutions, a small percentage of glycerine and alcohol were added, to affect the solution, but not enough to materially increase the antiseptic power. The temperature of the room was kept between 60° and 75° Farenheit.

| Strength of Thymol Solution.... | $\frac{1}{8000}$ | $\frac{1}{7000}$ | $\frac{1}{6000}$ | $\frac{1}{5000}$ | $\frac{1}{4000}$ | $\frac{1}{3000}$ | $\frac{1}{2000}$ | $\frac{1}{1000}$ | $\frac{1}{500}$ . | Simple Hydrant Water. |
|---|---|---|---|---|---|---|---|---|---|---|
| Bacteria present... | 2d day | 2d day | 2d day | 2d day | 2d day | 2d day | 2d day | 3d day | 4th day | 1st day. |
| Putrid odor began. | 2d day | 2d day | 2d day | 2d day | 2d day | 2d day | 3d day | 4th day | No odor in ten days | 2d day. |

A glance at the table shows that instead of being efficient in the strength of $\frac{1}{5000}$, it required $\frac{1}{500}$, or a solution of ten times the former strength, to prevent the development of putrid odor, and even in that solution, living bacteria were found by the microscope. A carbolic acid solution of about the same strength, showed on the tenth day neither bacteria, nor any putrid odor. It is evident from these results that thymol is inferior in power to carbolic acid, instead of being immensely superior.

Inspection of the table shows, however, that an antiseptic may retard putrefaction, and thus be somewhat useful, though not thoroughly efficient. Thus a solution of thymol of the strength of $\frac{1}{2000}$ retarded the development of putrid odor until the third day.

The actual surgical use of an antiseptic requires preparations stronger than the minimum solution found efficient in vial or bottle. A solution of one part of thymol in two thousand for instance, has a slight antiseptic power, but when injected into the complex cavity of a putrescent lumbar abscess, it is further diluted by the secretions within, and such small portions as are at last forced into the deeper recesses, are mingled with so large a quantity of putrid pus as to become wholly inefficient. Clinical experience shows that for the purification of such cavities, carbolic acid is required in the strength of at least one part in forty, and thymol should be still stronger, though the quantity can be diminished after a thorough depuration of the whole sac has once been effected.

These experiments had already been made by me before I learned that the Imperial Board of Health of Germany had recently completed a very exhaustive investigation of all the principal antiseptics. Their report shows that thymol, instead of being a very strong, is actually one of the very weakest of all antiseptics. They grant it, however, to be somewhat effective in the strength of one part in two thousand. As this report will become a standard authority in the world, I wish to caution the profession against trusting in surgical practice in the minimum strength found effective in laboratory experiments. For the reasons given, the complex pockets of a lumbar abscess, or of a suppurating knee joint, will not be disinfected by injecting minimum solutions.

In a recent case which fell under my advice, a lumbar abscess was held in perfect subjection for months, by forced injections of one part of carbolic acid to forty of water. Circumstances having placed the patient temporarily under the care of a practitioner who had faith in weak solutions, septic action was promptly set up, and in two weeks the man was in the full enjoyment of hectic fever, and of a putrid discharge from the abscess. Fortunately he returned in time within my reach, and a few days of forced injection of carbolic acid, at first in the proportion of one part to thirty and afterward of one to forty, arrested the mischief both locally and constitutionally, and restored the patient to comfort and safety.

NO. 6 SIXTEENTH ST., CHICAGO.

